# Possible neurocognitive benefits of exercise in persons with heart failure

**DOI:** 10.1186/s11556-015-0151-x

**Published:** 2015-10-23

**Authors:** Rachel Galioto, Andrew F. Fedor, John Gunstad

**Affiliations:** Department of Psychology Sciences, Kent State University, Kent, OH 44242 USA

**Keywords:** Heart failure, Exercise, Memory

## Abstract

More than 6 million Americans have heart failure (HF) and more than 500,000 are diagnosed each year. In addition to its many adverse medical consequences, HF is also a significant risk factor for neurological disorders like Alzheimer’s disease and associated with cognitive impairment long prior to the onset of these conditions. Converging bodies of literature suggest cognitive dysfunction in HF may be at least partially modifiable. One key mechanism for cognitive improvement is improved cerebral blood flow, which may be possible with exercise in patients with HF. This brief review provides a model for the likely neurocognitive benefits of exercise in HF and encourages further work in this area.

## Introduction

Heart failure (HF) has become an epidemic and nearly 6 million Americans have been diagnosed with this high risk condition [79]. Despite improved survival rates, the 5-year mortality rate remains at 50–60 % [61, 82]. HF also represents a significant individual and financial burden from high rates of rehospitalization and medications costs. HF is the most common reason for recurrent hospitalization and costs approximately $30 billion annually in the United States alone [35]. HF also produces significant psychosocial problems, including decreased functional independence and quality of life [1, 16].

### HF and neurocognitive function

In addition to medical and psychosocial consequences, HF is a significant risk factor for neurological disorders including Alzheimer’s disease, vascular dementia [75], and stroke [103, 104], and high rates of cognitive impairment even the absence of these conditions [97]. Recent studies show that the majority of individuals with HF evidence at least some cognitive impairment, while up to 25 % demonstrate moderate to severe cognitive impairment on testing [17]. Deficits have been observed in many different domains including attention, executive function, learning and memory, language, visuospatial functioning and psychomotor speed [6, 14, 17, 32, 74, 97, 98]. Interestingly, a recent study in HF patients found that nearly one quarter of the patients exhibited deficits in three or more domains of cognitive function [74]. The risk for cognitive dysfunction appears to increase with increasing HF severity [74, 97].

Cognitive dysfunction in HF is likely explained by a number of adverse brain changes that are also frequently observed in HF. Most commonly, patients demonstrate increased cortical atrophy [106], cerebral infarcts [4, 84], white matter changes [14] and metabolic alterations [60]. Specifically, patients with HF have been shown to have significantly less gray matter volume, especially in the insular cortex, frontal cortex, parrahippocampal gyrus, cingulate, cerebellar cortex and deep cerebellar nuclei [106] compared to controls. Additionally, HF patients exhibit increased amounts of periventricular white matter hyperintensities (WMH) and WMH in the basal ganglia [84, 99]. Other studies have found damage to the hippocampus, caudate nuclei, and the corpus callosum [105] and reduced mamillary body volume and cross-sectional areas of fornix fibers [58] in patients with HF.

Only a few studies have directly examined the association between the adverse brain changes and cognitive deficits observed in HF. Beer et al. [14] found that HF patients performed significantly worse than controls on visuospatial, executive functioning, visual memory and verbal learning tasks. Among these patients, left medial temporal lobe atrophy and deep WMH were significantly associated with impaired scores on measures of cognitive functioning. In another study, Vogels and colleagues [98] demonstrated that increased medial temporal lobe atrophy in patients with HF was associated with worse poorer performance on tests of memory, executive function and on the Mini Mental Status Exam independent of cardiovascular risk factors (e.g., hypertension).

## Review

### Can cognitive function be improved in HF?

The trajectory of cognitive impairment and possible decline in HF remains poorly understood. Despite being a known risk factor for degenerative disorders like Alzheimer’s disease and vascular dementia (e.g., [75]), two recent studies found that cognitive function remains relatively stable over short time intervals in patients with mild HF ([6, 78]). Moreover, there is research to suggest that the cognitive deficits of HF may be at least partly reversible. For example, a sample of 40 well-managed HF patients showed subtle improvements in cognitive function over a 12 month period, particularly in the areas of attention and executive function [87]. Though the exact mechanisms for these cognitive gains are unclear, it appears most likely attributable to improved medical oversight for the study participants [87]. Similarly, other studies have shown improved cognitive function in persons with HF as a result of medical intervention, including cardiac transplantation [17, 20, 43, 66] pacemaker and cardiac assist device implantation [73, 108], and initiation of treatment with ACE inhibitors [7, 109]. In each case, improved cardiac function was associated with better cognitive function after treatment. Taken together, these results suggest that cognitive impairment in HF may be at least partially reversible through improved cardiovascular function.

### Can exercise improve cognitive function in HF?

Exercise interventions have been linked to improved neurocognitive outcomes across a wide range of patient and healthy samples [29, 71]. Aerobic exercise is linked to greater gray and white matter volume [30] and increased functional connectivity in the prefrontal cortex [102]. The most consistent effects of aerobic exercise on cognition have been in executive functioning, although several investigations have found improvements in other domains such as attention, visuospatial functioning, processing speed [3, 18, 36]. For example, Voss et al. [101] demonstrated that one-year of exercise training was associated with improved working memory performance in healthy older adults. Even exercise at low intensities has been shown to improve attention [45], memory [81], and concentration [89] in healthy older adults.

### Mechanisms for cognitive improvement with exercise

Improvements in cognitive function with exercise are likely related to beneficial brain changes. For example, research has shown that increased cardiorespiratory fitness is associated with reduced brain atrophy [29], the preservation of gray and white matter in the medial-temporal, parietal, and frontal brain regions ([80]), and greater hippocampal volumes [38]. Higher fitness levels have also shown positive effects on functional brain outcomes including greater activation in areas associated with attentional control [31] and greater activity in the frontal and parietal lobes [30]. Moderate- to high- intensity aerobic exercise has produced similar benefits including increases in gray and white matter volume [30] and increased functional connectivity in the prefrontal cortex [102].

Exercise may improve cognitive function in HF patients through other mechanisms. For example levels of C-reactive protein (CRP), normally an inflammatory cytokine associated with acute injury [72], are inversely related to amount of physical activity ([28]; [25, 59]). Exercise is thought to reduce activation of the sympathetic nervous system, which in turn inhibits the release of inflammatory markers, including CRP [28]. This hypothesis has some support in the literature with a heart failure population. Following 6 months of structured exercise, HF patients demonstrated significantly lower levels of CRP, than sedentary controls [68]. The lower levels of CRP may also be related to cognitive function. Research suggests increased levels of CRP are related to impairments in the areas of executive function and memory [50, 70, 93, 107].

Prior work has also identified various circulating biomarkers which may also influence cognitive function in HF. There is little work done on these markers in relation to cardiovascular fitness as most are either associated with eating behavior or newly discovered themselves (i.e. adiponectin). In light of these shortcomings, some research has been conducted examining the influence of physical exercise on biomarkers. Brain derived neurotrophic factor (BDNF) has demonstrated positive relationship with exercise [42, 53]. This relationship has also been found in an HF population [39], and is important as research indicates cognitive impairment is at least partially caused by decreased BDNF levels [10]. Additionally, BDNF is important for brain health and cognitive function (e.g., [12, 64]).

Leptin has also been connected to cognitive function [62]. Specifically, leptin has been inversely related to level of cardiovascular fitness levels in both HF [90] and non-HF populations [21, 77]. Ghrelin, is a largely under researched hormone, thus, little evidence exists in relation to cardiovascular fitness. However, one study found ghrelin to have an inverse relationship with cardiovascular fitness [86]. Finally, adiponectin has also been studied in relation to cardiovascular fitness as well. Improvements in cardiovascular fitness have been associated with reduced adiponectin levels [11, 65].

In HF, improved cognitive performance with exercise may also be related to comorbid medical conditions. HF is associated with several cardiac and non-cardiac comorbidities; up to 40 % of HF patients have at least five non-cardiac medical conditions [22]. The presence of these comorbid conditions in patients with HF is associated with decreased quality of life, poorer prognosis [67], increased rates of hospitalization, and higher rates of mortality [22]. Common comorbidities of HF include hypertension, type 2 diabetes mellitus, obstructive sleep apnea, chronic obstructive pulmonary disorder, and depression. Each of these conditions has been shown to have an independent association with cognitive deficits, either in HF or non-HF populations, and are likely add to or interact with cardiac dysfunction in HF [49]. Exercise is a common non-pharmacological treatment for a number of comorbid conditions and has been shown to prevent the development or reduce the severity of such conditions both in HF and non-HF populations (e.g., [9, 19, 23, 57, 69]).

### Cerebral blood flow as a mechanism for cognitive improvement with exercise in heart failure

One key mechanism for cognitive gains with exercise which may be particularly important in HF patients is improved cerebral blood flow (CBF). Patients with HF show up to a 30 % reduction in global cerebral blood flow (CBF) [43]. Typically, CBF reductions appear to be greatest in posterior cortical areas [8] but have also been observed in other brain regions important for cognitive function including the frontal, temporal, and parietal lobes [8, 24, 100]. Reduced CBF is also related to poorer cognitive function in HF. In one study, resting regional CBF in elderly patients with HF was compared to healthy age-matched controls using single-photon emission computed tomography (SPECT). Results of this study demonstrated that reduced CBF was common in patients with HF and associated with poorer global cognition, visual and verbal memory, learning, and language tests. Importantly, global cognition was significantly associated with CBF in the posterior cingulate cortex and precuneus [8]. Another study found that global cognition, measured by performance on the Mini Mental Status Exam (MMSE), was significantly positively associated with CBF velocity of the right middle cerebral artery (MCA) in patients with HF [51].

### Increased CBF is associated with improved cognitive function in patients with HF

Intervention studies have shown that increased CBF is linked to improvements in cognitive function in HF. As above, many of the HF treatments that have been shown to improve cognitive function (e.g., cardiac transplantation, pacemaker implantation, ACE inhibitors) are also known to improve CBF [27, 43, 66]. Several studies have shown that although CBF is reduced at baseline, they become normalized following cardiac transplantation representing an increase of up to 30 % [27, 43, 66]. Similar effects have been observed following implantation of a pacemaker [96]. Finally, in patients with severe HF, CBF improved by approximately 12 ml/100 g per minute following the initiation of treatment with an ACE inhibitor and normalized over time [76]. Given that HF treatment such as cardiac transplantation, pacemaker implantation and ACE inhibitors have been shown to both improve cognitive function and increase CBF, it can be reasoned that increases in CBF may be an important mechanism for improved cognitive function in HF patients.

### Can exercise improve CBF and cognitive function in HF?

#### Evidence for improved CBF with exercise

**Fig. 1:**
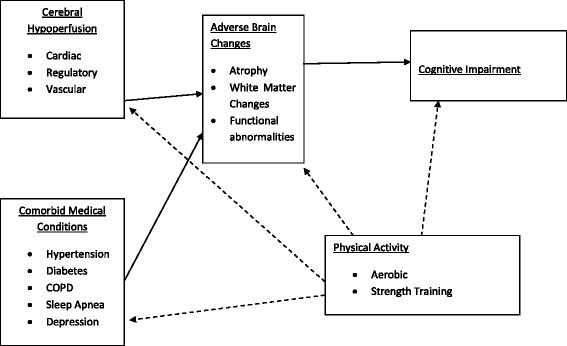
Physical Activity for the Improvement of Cognitive function in Heart Failure Solid lines represent pathways leading to poorer health and cognitive outcomes. Broken lines indicate pathways for improved outcomes

Reduced CBF in HF is, in part, the result of decreased cardiac, regulatory, and vascular functioning. In particular, it appears that the combination of reduced cardiac output (CO) and [83], decreased cerebral autoregulation [46], and impaired endothelial functioning [48] lead to decreased cerebral perfusion and ischemic damage in patients with HF. Importantly, exercise has been shown to improve cardiac and vascular function [41, 44] in HF patients potentially leading to increased CBF. See Fig. 1.

Moderate- to high-intensity aerobic exercise has been shown to improve exercise capacity and increase VO_2_ max in patients with HF [34, 52, 54, 55] and is also associated with a number of cardiac and vascular improvements among patients with HF. In terms of cardiac functioning, the benefits of moderate- to high intensity aerobic exercise include decreased resting HR [34, 37, 44, 85], increased CO [44, 92] and stroke volume [34, 37, 44] and reduced resting LV end-diastolic diameter [44]. In terms of vascular functioning, benefits include decreased peripheral resistance and sympathetic activation [44], increased vasodilatory capacity [63], blood flow [44] and improved endothelial function [63].

Exercise at lower intensities is also related to improved VO_2_ max and increased exercise capacity [15, 33, 56, 91] though research on its association with other cardiac and vascular factors is limited. One study also demonstrated that moderate-intensity (50 % max work rate) cycling was associated with improved HR recovery while participants who completed high-intensity interval training did not experience such improvement [33].

A growing body of literature shows aerobic exercise has beneficial effects on CBF in non-HF populations [2, 47]. Specifically, Hellstrom et al. [47] demonstrated that global CBF increased during moderate exercise in a sample of healthy adults. Another study found higher blood flow velocity in the middle cerebral artery among endurance-trained men when compared to sedentary men [2]. Similarly, a recent study demonstrated higher resting CBF levels among older master athletes when compared to sedentary older adults [95]. It has also been demonstrated that 12 weeks of aerobic exercise was associated with both improved CBF and cognition in healthy older adults [26]. Although no study to date has examined whether exercise can improve CBF in patients with HF, one study has examined this association in a sample of older adults with cardiovascular disease (CVD) [88]. In this study, 12 weeks of exercise was associated with improved CBF velocity. The authors also found that attention, executive function, and memory performance improved, though these improvements were not related to CBF velocity.

### Evidence for cognitive improvement with exercise in HF

There has been some research to suggest that cognition can improve following exercise in HF. For example, Tanne et al. [94] examined the benefits of twice weekly aerobic exercise at 60–70 % of maximal heart rate on cognitive function in HF patients. Results demonstrated that exercise was associated with improvements in attention/psychomotor speed and executive function. Unfortunately, these findings are limited by a small number of participants in the intervention (*n* = 18) and control group (*n* = 5) and potential baseline differences in cognitive function between these groups were not examined. Additionally, CBF was not measured.

Consistent with these possible benefits of exercise, two recent studies have examined the link between fitness levels and cognitive function in HF. One study found that greater metabolic equivalents (METs) from a standardized stress test was related to better performance on measures of attention (β = .41, *p* = .03), executive function (β = .37, *p =* .04), and memory (β = .46, *p* = .04) even after controlling for important medical and demographic characteristics, [40]. Similarly, another study examined the association between exercise capacity, estimated by distance walked on the 6-min walk test, and cognitive function in 80 elderly patients with HF. As above, results showed that greater exercise capacity was associated with better cognitive function [13].

## Conclusion

Overall, the current evidence seems to suggest that the cognitive benefits through exercise could extend to persons with HF. In particular, findings from interventional studies (i.e., pacemaker implant, cardiac transplant, treatment with ACE inhibitors) suggest that improved CBF can lead to improved cognitive functioning in patients with HF. Exercise may also lead to similar improvements through its beneficial effects on cardiac and vascular functioning in HF patients, potentially leading to improved CBF and ultimately, improved cognitive function. Existing research on the cognitive benefits of exercise in HF is limited, but promising. The search for interventions that can improve cognitive functioning or prevent further decline in patients with HF are much needed, as the societal implications of such an intervention would be substantial.
